# Maternal milk fat globule membrane enriched gut *L. murinus* and circulating SCFAs to improve placental efficiency and fetal development in intrauterine growth restricted mice model

**DOI:** 10.1080/19490976.2024.2449095

**Published:** 2025-01-06

**Authors:** Cuiping Feng, Yujun Wu, Xiangyu Zhang, Shuxian Wang, Junjun Wang, Huixia Yang

**Affiliations:** a Department of Obstetrics and Gynecology and Reproductive Medicine, Peking University First Hospital, Beijing, China; b Department of Obstetrics and Gynecology, China-Japan Friendship Hospital, Beijing, China; c State Key Laboratory of Animal Nutrition and Feeding, College of Animal Science and Technology, China Agricultural University, Beijing, China

**Keywords:** Intrauterine growth restriction, *Lactobacillus murinus*, milk fat globule membrane, placental functions, short chain fatty acids

## Abstract

Intrauterine growth restriction (IUGR) caused by placental dysfunctions leads to fetal growth defects. Maternal microbiome and its metabolites have been reported to promote placental development. Milk fat globule membrane (MFGM) is known for its diverse bioactive functions, while the effects of gestational MFGM supplementation on the maternal gut microbiota, placental efficiency, and fetal development remained unclear. In this study, low protein diet-induced IUGR decreased the litter birth weight, fetal birth weight, and the fetal/placental ratio in pregnant mice, while gestational MFGM supplementation restored these impairments. Meanwhile, MFGM supplementation during gestation enriched intestinal *Lactobacillus murinus* (*L. murinus*) and increased luminal and circulating short chain fatty acids (SCFAs) in IUGR pregnant mice, which improved placental efficiency and fetal development due to an enhanced antioxidant capacity and a decreased inflammation. In addition, fecal microbiota transplantation (FMT) with MFGM-derived microbiota reprinted the promoted phenotypes of maternal litter characteristics, gut *L. murinus* enrichment, placental efficiency, and fetal gut development in MFGM-fed pregnant mice, which were also recapitulated by exogenous administration with *L. murinus* or SCFAs cocktail. Mechanically, MFGM, MFGM-derived microbiota, *L. murinus*, or SCFAs cocktail activated IUGR-induced depressive phosphorylation of PI3K-Akt signaling in the placenta. Moreover, *in vitro* placental cells cultivation under amino acid shortage model (AAS) or oxygen-glucose shortage model (OGS) was used to validate that MFGM-derived key microbial and circulating SCFAs cocktails can alleviate placental oxidative stress and inflammation via activating PI3K/Akt signaling. Taken together, gestational MFGM supplementation enriched intestinal *L. murinus* and circulating SCFAs of IUGR pregnant mice, thereby improving placental efficiency, fetal growth, and intestinal functions of IUGR fetus. Our findings will provide theoretical support for the application of MFGM in the maternal–placental-fetal nutrition to address pregnancy malnutrition-induced IUGR.

## Introduction

Intrauterine growth restriction (IUGR), also known as fetal growth restriction (FGR), refers to the impaired growth and development of the mammalian embryo/fetus during pregnancy.^1^ It has become a common public health concern, with a prevalence of up to 30% in developing countries and 8% in developed countries.^2^ IUGR increases the risk of perinatal mortality and morbidity, along with a series of short- and long-term adverse health outcomes.^3^ Our previous findings showed that IUGR can induce immune dysfunction and intestinal barrier impairment in neonatal piglets, leading to life-long adverse impact on the intestinal health and development.^4,5^ The occurrence of IUGR can be associated with placental inefficiency, including reduced blood sinusoid area and oxidative damage.^6^ Therefore, it is of great importance to develop effective strategies to improve placental efficiency of IUGR maternities, growth performance, and intestinal functions of IUGR fetus.

Maternal intestinal microbiota and its metabolites, which can have critical impacts on fetal intestine and placenta development, vary greatly between normal and IUGR maternities and neonates.^7–10^ *Lactobacillus* has been reported as a critical probiotic with variable benefits for maternal health and fetal development.^11,12^ Our previous study indicated that maternal circulating short chain fatty acids (SCFAs), as key microbial metabolites with large bioactive properties, were negatively associated with immune-metabolic process within placenta, suggesting a critical role of SCFAs in regulating placental functions and FGR fetus development.^13^ Thus, modulation of the intestinal microbiota and their metabolites was one of the potential targets to promote maternal-placental-fetal health outcomes.

Milk fat globule membrane (MFGM), composed of membrane-specific phospholipids, sphingolipids, and proteins, has been identified as a substance possessing physiological and functional properties in regulating immune response and improving intestinal development.^14–16^ It is reported that MFGM is capable of alleviating LPS-induced intestinal inflammation and improve antioxidant capacity in neonatal IUGR mice.^17^ Our previous results indicated that supplementing MFGM to gestating sows reduced the number of stillbirths at farrowing and improved intestinal development of their offspring.^18^ However, the effects and underlying mechanisms of gestational MFGM intervention on maternal microbial composition, placental efficiency, and fetal development under IUGR status remained largely unclear.

Here, we hypothesized that MFGM intervention during gestation will modulate maternal gut microbial composition, improve placental functions and fetal development, thereby promoting the IUGR maternal-placental-fetal health outcomes. This study aimed to investigate the effects and underlying mechanisms of maternal MFGM supplementation on the placental functions and fetal growth, based on a low protein diet-induced IUGR pregnant mice model, amino acid shortage or oxygen-glucose shortage-induced placental cell malnutrition model.

## Materials and methods

### Ethics statement

All the animal treatments and procedures were performed with the approved protocols by China Agricultural University Animal Care and Use Committee (AW13014202-1-4, Beijing, China).

### Establishment of IUGR pregnant mice model

Eight-week-old male and female mice were purchased from SPF (Beijing) Biotechnology Co., Ltd. (Beijing, China) and housed under identical conditions with a 12 h light (L):12 h dark (D) photoperiod at 23 ± 2°C, with free access to feed and water. After female mice mated (1:1) overnight, the detection of vaginal plug was considered as day 0.5 of gestation (GD0.5) and then, the pregnant mice were housed individually in separate cages. IUGR pregnant mice model was established according to a previous study.^19^ Briefly, mice were fed a normal protein diet (NP) before GD10.5 and a low protein diet (LP) from GD10.5 to GD18.5. The nutritional compositions of NP diet and LP diet were shown in Supplementary Table S1.

### Experimental treatments for IUGR pregnant mice

**Figure 1.**
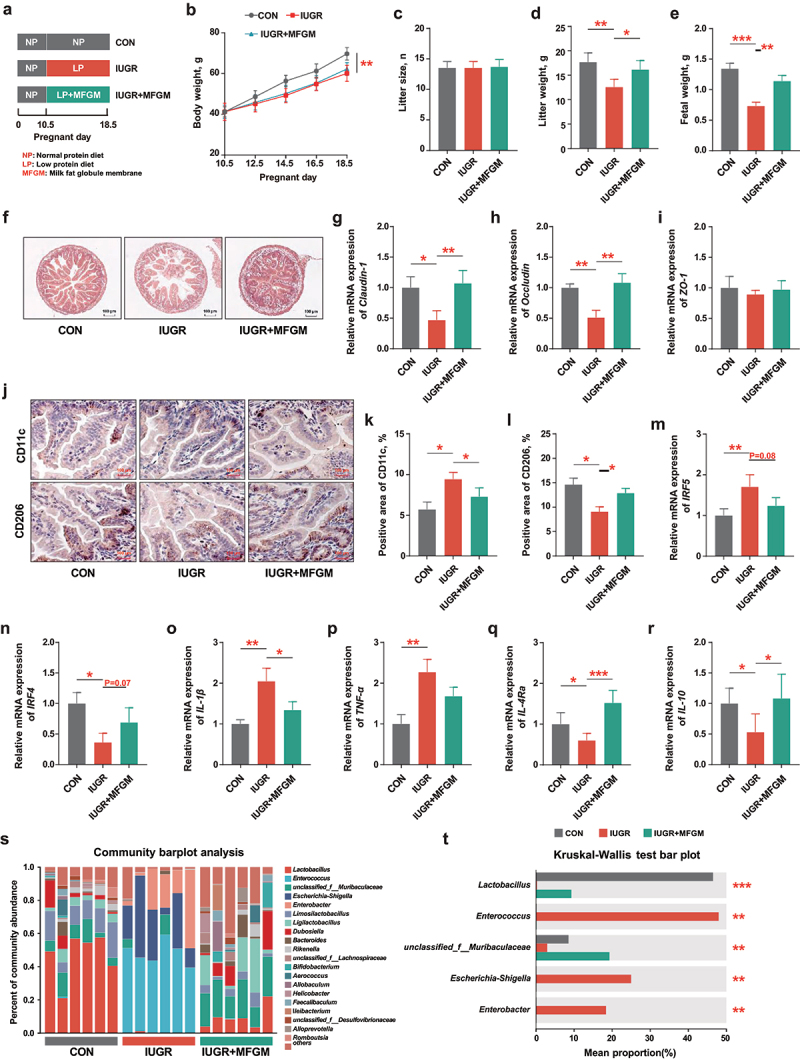
After mated, 8-week-old pregnant C57BL/6J mice (*n* = 6 per group) were fed with a normal protein diet (NP) before gestational day (GD) 10.5 and a low protein diet (LP) from GD10.5 to GD18.5 to induce intrauterine growth restriction (IUGR) and supplemented with/without MFGM (400 mg/kg BW). (a) Study design; (b-e) body weight, litter size, litter weight and fetal weight of pregnant mice; (f-i) morphology and relative gene expressions of tight junctions Claudin-1, Occludin and ZO-1 of fetal jejunum; (j-n) immunohistochemical staining, percentage and relative gene expressions of biomarkers of M1/M2 macrophages in fetal jejunum; (o-r) relative gene expression of inflammatory cytokines of fetal jejunum; (s,t) fecal microbial analysis of community plots and differential microbiota of pregnant mice. *n* = 6; the Kruskal–Wallis test and post-hoc Tukey–Kramer test was used for microbial analysis, and one-way ANOVA with Tukey’s test was used for statistical analysis of all other parameters. *p < 0.05, **p < 0.01, ***p < 0.001. CON, mice fed with normal protein diet from G0.5-G18.5; IUGR, mice fed with normal protein diet before G10.5 and low protein diet from G10.5-G18.5; IUGR + MFGM, mice fed with normal protein diet before G10.5 and low protein diet supplemented with MFGM from G10.5-G18.5; IRF4, interferon regulatory factor 4; IRF5, interferon regulatory factor 5; IL-10, interleukin-10; IL-4 Rα, interleukin-4 Rα; tnf-α, tumor necrosis factor-α; IL-1β, interleukin-1β; ZO-1, zonula occludens-1.

For MFGM administration, a total of 18 pregnant Institute of Cancer Research (ICR) mice (G10.5) were randomly divided into three groups (Figure 1a, *n* = 6): (1) Control (CON), fed with a standard diet and gavaged with phosphate-buffered saline (PBS) daily; (2) IUGR, fed with LP diet and gavaged with PBS daily; (3) IUGR + MFGM, fed with LP diet and gavaged with MFGM solution daily (400 mg/kg bodyweight). The dose referred to a previous study.^20^ MFGM was provided by Beijing Sanyuan Foods Co. Ltd (Beijing, China), and the composition was shown in Supplementary Table S2.

**Figure 2.**
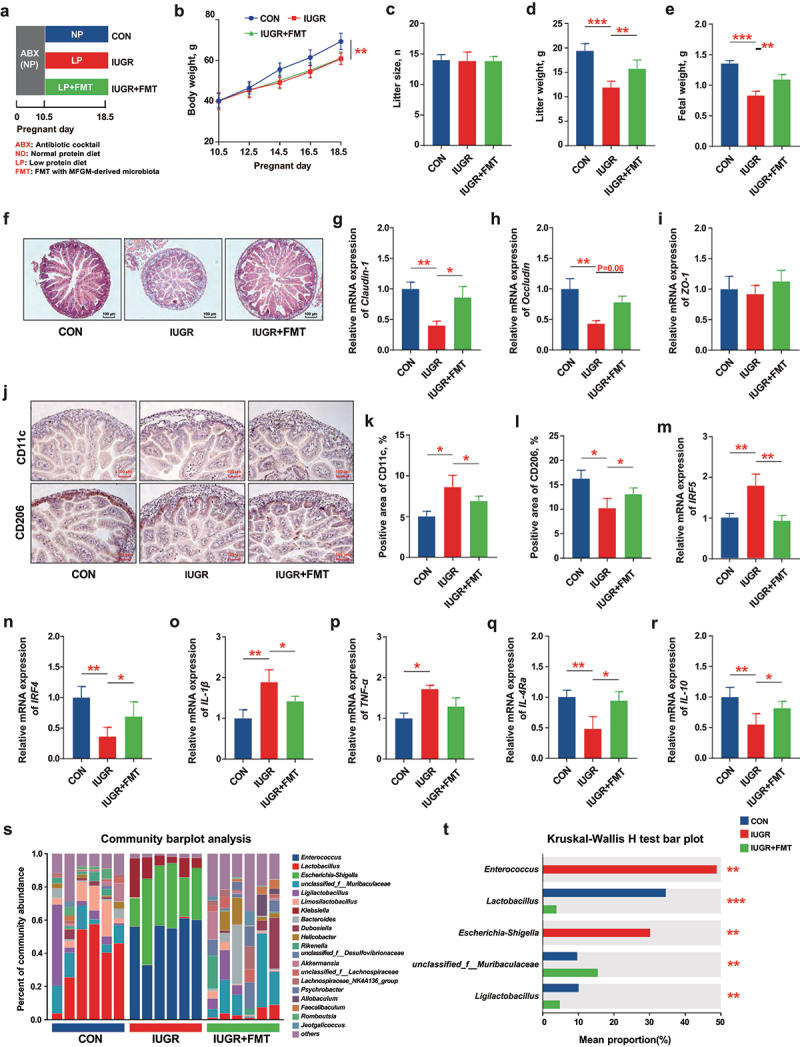
*Lactobacillus* was enriched in the receipt mice by fecal microbiota transplantation (FMT) with mfgm-derived microbiota. After mated, 8-week-old male C57BL/6J mice (*n* = 6 per group) were fed with a normal protein diet (NP), and the intestinal microbiota were depleted with a cocktail of antibiotics before gestational day (GD) 10.5 and a low protein diet (LP) from GD10.5 to GD18.5 to induce intrauterine growth restriction (IUGR) and administered with/without MFGM-derived fecal microbial suspension (0.2 mL/d). (a) Study design; (b-e) body weight, litter size, litter weight and fetal weight of pregnant mice; (f-i) morphology and relative gene expressions of tight junctions Claudin-1, Occludin and ZO-1 of fetal jejunum; (j-n) immunohistochemical staining, percentage and relative gene expressions of biomarkers of M1/M2 macrophages in fetal jejunum; (o-r) relative gene expression of inflammatory cytokines of fetal jejunum; (s,t) fecal microbial analysis of community plots and differential microbiota for pregnant mice. *n* = 6; the Kruskal–Wallis test and post-hoc Tukey–Kramer test was used for microbial analysis, and one-way ANOVA with Tukey’s test was used for statistical analysis of all other parameters.* *p* < 0.05, ** *p* < 0.01, *** *p* < 0.001. CON, mice fed with normal protein diet from G0.5-G18.5; IUGR, mice fed with normal protein diet before G10.5 and low protein diet from G10.5-G18.5; IUGR + FMT, mice fed with normal protein diet before G10.5 and low protein diet plus FMT with MFGM-derived fecal microbial suspension from G10.5-G18.5; IRF4, interferon regulatory factor 4; IRF5, interferon regulatory factor 5; IL-10, interleukin-10; IL-4 Rα, interleukin-4 Rα; TNF-α, tumor necrosis factor-α; IL-1β, interleukin-1β; ZO-1, zonula occludens-1.

For fecal microbiota transplantation (FMT), a total of 18 pregnant ICR mice were treated with an antibiotic cocktail (ABX, 1 g/L streptomycin, 0.5 g/L ampicillin, 0.5 g/L vancomycin, and 1 g/L gentamicin) added to their drinking water for 10.5 d after mating to deplete intestinal microbiota, and the ABX water was refreshed every three days as described in a previous study.^21^ At G10.5, pregnant mice were randomly divided into three groups (Figure 2a, *n* = 6): (1) Control (CON), fed with a standard diet and gavaged with PBS daily; (2) IUGR, fed with an LP diet and gavaged with PBS daily; (3) IUGR + FMT, fed with an LP diet and gavaged with MFGM-derived fecal suspension daily (0.2 mL/d). The fecal suspension was obtained by homogenizing 100 mg feces from MFGM-fed mice into 1.5 mL sterile anaerobic PBS. The handling procedure and dose of fecal suspension referred to a previous study.^21^

**Figure 3.**
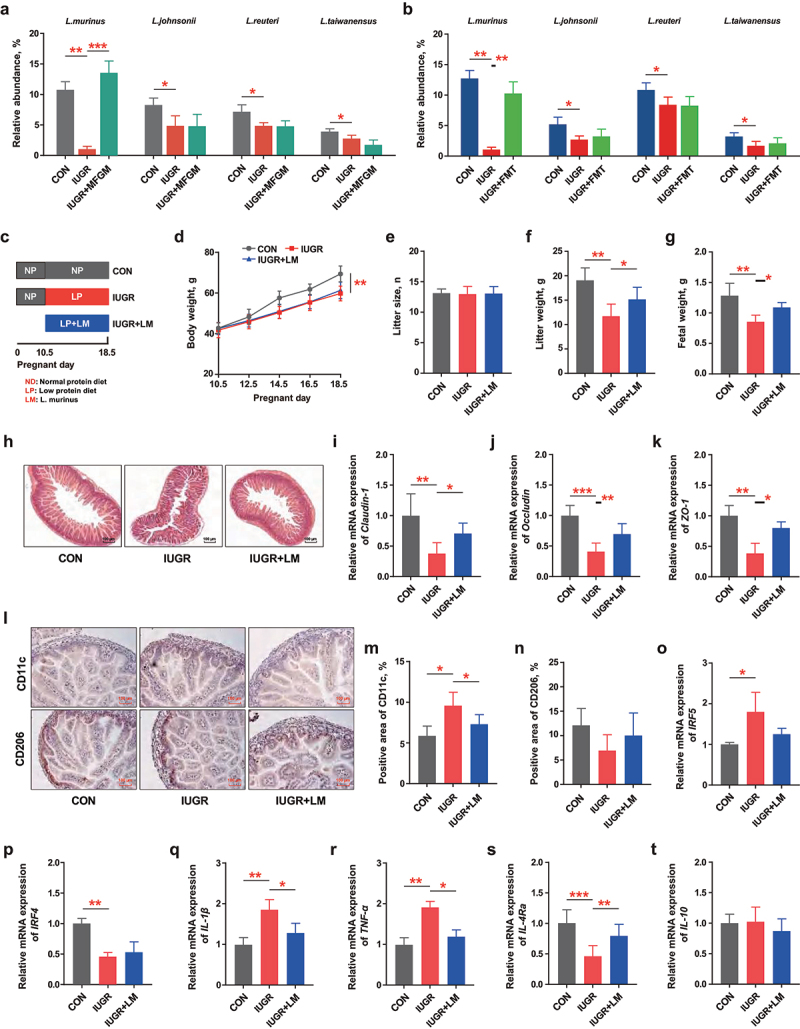
*L. murinus* administration improved the litter weight and fetal development of IUGR pregnant mice. (a, b) Relative abundance of *Lactobacillus* species in the feces of IUGR pregnant mice supplemented with/without MFGM or MFGM-derived fecal microbial suspension; after mated, 8-week-old pregnant C57BL/6J mice (*n* = 6 per group) were fed with a normal protein diet (NP) before gestational day (GD) 10.5 and a low protein diet (LP) from GD10.5 to GD18.5 to induce intrauterine growth restriction (IUGR) and administered with/without *L.* *murinus* (2 × 10^8^ CFU/d). (c) Study design; (d-g) body weight, litter size, litter weight and fetal weight of pregnant mice; (h-k) morphology and relative gene expressions of tight junctions Claudin-1, Occludin and ZO-1 of fetal jejunum; (l-p) immunohistochemical staining, percentage and relative gene expressions of biomarkers of M1/M2 macrophages in fetal jejunum; (q-t) relative gene expression of inflammatory cytokines of fetal jejunum. *n* = 6; one-way ANOVA with Tukey’s test was used for statistical analysis. *p < 0.05, **p < 0.01, ***p < 0.001. CON, mice fed with normal protein diet from G0.5-G18.5; IUGR, mice fed with normal protein diet before G10.5 and low protein diet from G 10.5-G18.5; IUGR + LM, mice fed with normal protein diet before G10.5 and low protein diet plus *L.* *murinus* administration from G10.5-G18.5; LM, *L.* *murinus*; IRF4, interferon regulatory factor 4; IRF5, interferon regulatory factor 5; IL-10, interleukin-10; IL-4 Rα, interleukin-4 Rα; TNF-α, tumor necrosis factor-α; IL-1β, interleukin-1β; ZO-1, zonula occludens-1.

For *Lactobacillus murinus* (LM) administration, a total of 18 pregnant ICR mice (G10.5) were randomly divided into three groups (Figure 3a, *n* = 6): (1) Control (CON), fed with a standard diet and gavaged with PBS daily; (2) IUGR, fed with an LP diet and gavaged with PBS daily; (3) IUGR + LM, fed with an LP diet and gavaged with *L. murinus* daily (2 × 10^8^ CFU/d). The dose of *L. murinus* referred to a previous study.^22^

For SCFAs cocktail administration, 18 pregnant ICR mice (G10.5) were randomly divided into three groups (Figure 5a, *n* = 6): (1) Control (CON), fed with a standard diet and gavaged with PBS daily; (2) IUGR, fed with an LP diet and gavaged with PBS daily; (3) IUGR + SCFA, fed with an LP diet and administered with SCFAs cocktail in drinking water. The SCFAs cocktail contained a mixture of sodium acetate (67.5 mM), sodium propionate (25.9 mM), and sodium butyrate (40 mM). The composition and dose of SCFAs cocktail referred to a previous study.^23^

For data collection and sampling, maternal body weight was recorded every other day. At G18.5, feces were collected from the pregnant mice. After that, pregnant mice were anesthetized and underwent blood collection and cesarean section. Serum was obtained by centrifugation with 3,000 rpm for 10 min. Placenta weight, fetal number, and weight were individually measured. One placenta and fetal jejunum per maternal mouse were randomly selected (*n* = 6 per group) and fixed in 4% paraformaldehyde. Other placenta and jejunum samples were snap-frozen and stored at −80°C for further analysis.

### Isolation and culture of *L.* *murinus*

Fecal samples from pregnant mice were anaerobically suspended in sterile PBS buffer to create bacterial suspension with a tenfold gradient. After each dilution (100 μL) anaerobically spread on De Man, Rogosa, and Sharp medium (MRS) agar medium and incubated at 37°C for 24 h, single colonies were anaerobically transferred into broth (5 mL) and incubated at 37°C for 24 h. After that, the bacterial total DNA was extracted and amplified by polymerase chain reaction (PCR) with the universal primers 27F (AGRGTTYGATYMTGGCTCAG) / 1492R (RGYTACCTTGTTACGACTT). The PCR product was sequenced and then scanned the National Center for Biotechnology Information (NCBI) nucleotide sequence database to identify *L. murinus*. The identified *L. murinus* was stored in 25% sterile glycerol at −80°C for further experiment.

### Cells culture and treatment

Human choriocarcinoma originated BeWo cell line was purchased from the National Infrastructure of Cell Line Resource (China) and maintained in Dulbecco’s Modified Eagle Medium F-12 (DMEM/F12) medium supplemented with 10% fetal bovine serum (FBS), 100 U/mL penicillin, 100 U/mL streptomycin, and 250 ng/mL amphotericin B at 37°C and 5% CO_2_. The syncytialization of BeWo cells was induced by incubation with 20 µM forskolin (FSK, Selleck, USA) for 48 h.

For amino acid shortage (AAS) model, BeWo cells were divided into three groups (Figure 7a, *n* = 3): (1) Control (CON), incubated with basic DMEM/F12 medium; (2) Amino acids shortage (AAS), incubated with amino acid shortage medium (AAS); (3) AAS+SCFA, incubated with AAS supplemented with SCFAs cocktail. The AAS medium consists of a 7:1 mixture of DMEM depleted of amino acids glutamine, lysine, and arginine (Gibco A1443101) and regular DMEM (Gibco 11965092) supplemented with 10% FBS. The 7:1 mixing ratio was based on a previous study.^24^

For oxygen‑glucose shortage (OGS) model, the BeWo cells were divided into three groups (Figure 7i, *n* = 3): (1) Control (CON), incubated with basic DMEM/F12 medium; (2) Oxygen-glucose shortage (OGS), incubated with glucose shortage medium in hypoxic conditions (94%N_2_/5%CO_2_/1%O_2_, OGS); (3) AAS+SCFA, incubated with OGS supplemented with SCFAs cocktail. The glucose medium consists of a 7:1 mixture of glucose-free DMEM (Gibco 11966025) and regular DMEM (Gibco 11965092) supplemented with 10% FBS.

The SCFAs cocktail contained a mixture of sodium acetate (6.75 µM), sodium propionate (2.59 µM), and sodium butyrate (4 µM). The dual inhibitor of PI3K/Akt, PI3K/AKT-IN-1 (MedChemExpress, China), was added into the AAS or OGS culture medium when needed. Cells were cultured for 24 h and harvested for further analysis.

### Hematoxylin & eosin staining (H&E)

Intestinal and placental samples were removed from 4% paraformaldehyde solution and dehydrated through a graded ethanol series (70% to 100%), then cleared with xylene and embedded in paraffin wax. Serial sections (5 μm thickness) were cut and stained with hematoxylin and eosin (H&E). Tissue sections were observed under a bright-field on a Zeiss Axio Imager microscope (Carl Zeiss Microscopy LLC, USA). The placental blood sinusoid area was analyzed by Image J (Media Cybernetics, USA).

### Immunohistochemical staining

The fetal intestinal tissues were dewaxed, rehydrated, irradiated, treated with 3% H_2_O_2_ for 10 min, blocked with normal goat serum, incubated overnight at 4°C with primary antibody (1:100), incubated at 37°C for 30 min with secondary antibody (CD11c, GB11059; CD206, GB13438), and stained by 3,3-diaminobenzidine (DAB). Finally, the slides were observed with a light microscope and quantified using Image Pro Plus software (version 6.0, Media Cybernetics, USA).

### DNA extraction, 16S rRNA sequencing, and data processing

Total genomic DNA of fecal samples were extracted using the QIAamp Fast DNA Stool Mini Kit (Qiagen, Germany) according to manufacturer’s instructions. The quality and concentration of DNA were determined using 1.0% agarose gel electrophoresis and a NanoDrop® ND-2000 spectrophotometer (Thermo Scientific Inc., USA). The V3-V4 regions of the 16S rRNA gene were amplified using universal primers 338F (ACTCCTACGGGAGGCAGCAG) and 806R (GGACTACHVGGGTWTCTAAT) and purified by AxyPrep DNA Gel Extraction Kit (Axygen Biosciences, USA). Then, the purified PCR products were pooled into equimolar amounts and sequenced on the Illumina NextSeq 2000 PE300 platform (Illumina, USA) according to the standard protocols by Majorbio Bio-Pharm Technology Co. Ltd. (Shanghai, China) to generate paired end reads of 300 bp.

Raw fastq files were de-multiplexed using an in-house perl script, and then quality-filtered by fastp (version 0.19.6) and merged by FLASH (version 1.2.11). The optimized sequences were clustered into operational taxonomic units (OTUs) using Usearch 11 with a 97% sequence similarity level. The most abundant sequence for each OTU was selected as a representative sequence. To minimize the effects of sequencing depth on alpha and beta diversity measure, the number of 16S rRNA gene sequences from each sample was rarefied to 20,000. The taxonomy of each OTU representative sequence was analyzed by RDP Classifier (version 2.13) against the 16S rRNA gene database (SILVA 138) using a confidence threshold of 0.7.

### SCFAs measurement by liquid chromatography tandem mass spectrometry (LC-MS/MS)

SCFA contents, including acetate, propionate, and butyrate, in plasma and feces of mice were measured by LC-MS/MS. Briefly, 100 μL plasma and 100 mg feces were thawed, and mixed with 400 μL or 1 mL cold methanol/acetonitrile (1:1, v/v) to remove the protein. After centrifugation (20 min 14,000 g, 4 C), the supernatants were freeze-dried and resolved in 100 μL acetonitrile/water (1:1, v/v) for LC-MS/MS analysis. Analysis was performed using a UHPLC (Agilent Technologies, USA) coupled with a QTRAP instrument (AB SCIEX, USA) using an ACQUITY UPLCBEH amide column (Waters MS Technologies, UK). MS/MS analysis was performed in the ESI negative mode. Data acquisition and processing were accomplished using Multiquant software (AB SCIEX, USA). Total SCFAs were the sum of acetate, propionate, and butyrate.

### RNA extraction, gene expressions detection by quantitative real-time PCR (qRT-PCR) of intestinal samples

Total RNA of intestinal samples was extracted using TRIzol reagent (Invitrogen, USA) according to the manufacturer’s instructions, and the cDNA was obtained using Prime Script^TM^ RT Kit (Takara, Japan). qRT-PCR was performed according to the SYBR Premix Ex Taq^TM^ II instructions (Takara, Japan) on a Light Cycler System (Roche, USA). Primers used for qRT-PCR were shown in Supplementary Table S3. Amplifications were performed in duplicates for each sample. The relative expressions of the target genes to the housekeeping gene β-actin were calculated according to the 2^−ΔΔCt^ method.^25^

### Transcriptome sequencing and analysis for placenta samples

Total RNA was extracted from the placenta for subsequent paired-end sequencing on the Illumina HiSeq platform to generate short reads of 150 bp. High-quality clean data were obtained by SeqPrep and Sickle with default parameters and then aligned to the *Mus_musculus* reference genome (GRCm39) in the orientation mode using HISAT2. Mapped reads were assembled by StringTie. Differentially expressed genes (DEGs) were identified through pairwise comparisons using DESeq2. DEGs with |log2 FC| > 1.5 and q < 0.05 were considered. Functional enrichment analyses of KEGG were performed at q < 0.05 using Goatools and KOBAS. Data analysis was performed on the Majorbio Cloud Platform (https://www.majorbio.com).

### Western blotting

Proteins were extracted using a radio immunoprecipitation assay (RIPA) buffer containing protease and phosphatase inhibitors and quantified using the bicinchoninic acid (BCA) method. Western blot was performed with the following antibodies: β-actin (Cell Signaling Technology, 13E5, 1:1,000), PI3K (Cell Signaling Technology, C73F8, 1:1,000), p-PI3K (Cell Signaling Technology, E3U1H, 1:1,000), Akt (Cell Signaling Technology, C67E7, 1:1,000), p-Akt (Cell Signaling Technology, D9E, 1:1,000), and HRP-conjugated secondary antibody (Cell Signaling Technology 91196, 1:10,000). The signal was visualized by FluorChem M (ProteinSimple, USA) and quantified using Image J software.

### Intracellular reactive oxygen species (ROS) detection

Intracellular ROS was measured by 2’,7’-dichlorofluorescein diacetate (DCFH-DA, in green) under manufacture’s instruction (YEASEN, China). Cells were washed and imaged with 488 nm excitation and 525 nm emission. The fluorescence intensity of was qualified with ImageJ software (version 20).

### Statistical analysis

The microbial analysis was performed on the free online platform of Majorbio Cloud platform (https://cloud.majorbio.com). Based on the OTUs information, the similarity among the microbial communities in different samples was determined by principal coordinate analysis (PCoA) based on unweighted unifrac distances using Vegan package (version 2.4.3). Kruskal-Wallis test and post-hoc Tukey-Kramer test were used to analyze bacterial α diversity and differentially enriched bacteria taxa. Spearman rank correlation coefficient was used for the correlation analysis of differential microbiota and other parameters.

Other data were analyzed by SPSS 20.0 (IBM, USA). One-way ANOVA with Tukey’s test was used for statistical analysis. All the results were considered significant at *p* < 0.05. GraphPad Prism (version 7, USA) was used for the graphical representations.

## Results

### MFGM supplementation enriched Lactobacillus and improved litter weight and fetal development of IUGR pregnant mice

To study the effects of MFGM on the litter characteristics and fetal development, a low protein diet was fed to pregnant mice (G10.5) with/without MFGM supplementation (Figure 1a). Results showed that MFGM supplementation improved the litter characteristics of IUGR pregnant mice by increasing litter weight (*p* < 0.05) and fetal weight (*p* < 0.01) without altering the maternal body weight or litter size (*p* > 0.05) (Figure 1b-e). Meanwhile, IUGR-induced fetal intestinal barrier dysfunction and immune imbalance were greatly ameliorated by maternal MFGM supplementation, evidenced by promoted intestinal morphology and upregulated tight junctions of *Claudin-1* (*p* < 0.01) and *Occludin* (*p* < 0.01) (Figure 1f-i). In addition, maternal MFGM supplementation decreased CD11c^+^ pro-inflammatory macrophages (*p* < 0.05) while increasing CD206^+^ anti-inflammatory macrophages (*p* < 0.05) in the fetal intestine, along with their biomarkers of *IRF4* (*p* = 0.07) and *IRF5* (*p* = 0.08), as well as cytokines expressions of *IL-1β* (*p* < 0.05), *IL-4Ra* (*p* < 0.001), and *IL-10* (*p* < 0.05) (Figure 1j-r). These results suggested that MFGM promoted maternal health and fetal development in IUGR pregnant mice.

To investigate the intestinal microbial composition of IUGR pregnant mice supplemented with MFGM, 16S rRNA sequencing of was conducted on maternal fecal microbiota. The results showed that the relative abundance of *Lactobacillus* (*p* < 0.001) and *unclassified_f__Muribaculaceae* (*p* < 0.01) were decreased, while *Enterococcus* (*p* < 0.01), *Escherichia-Shigella* (*p* < 0.01), and *Enterobacter* (*p* < 0.01) were increased in IUGR pregnant mice, which were reversed by MFGM supplementation (Figure 1s, t; Figure. S1A-C). These results suggested that MFGM-derived microbial alterations, especially the enrichment of *Lactobacillus*, might play a role in promoting maternal health and fetal development in IUGR mice model.

### Lactobacillus was enriched in the receipt mice by fecal microbiota transplantation (FMT) with MFGM-derived microbiota

To confirm the critical role of MFGM-derived gut microbiota in promoting maternal health and fetal development, FMT was performed in the microbial depleted receipt mice with antibiotic cocktail pretreatment (Figures 1a, S2). The results showed that FMT with MFGM-derived microbiota partially recapitulated the phenotype of MFGM by increasing litter weight (*p* < 0.01) and fetal weight (*p* < 0.01) of IUGR pregnant mice, without altering the maternal body weight and litter size (*p* > 0.05) (Figure 1b–e). Consistently, IUGR-induced fetal intestinal development was promoted by MFGM-derived microbiota with improved barrier and immune functions, characterized with better intestinal morphology, upregulated tight junctions of *Claudin-1* (*p* < 0.05) and *Occludin* (*p* = 0.06) (Figure 1f–i), and modulated macrophages polarization with their biomarkers of *IRF4* (*p* < 0.05) and *IRF5* (*p* < 0.01), along with cytokines expressions of *IL-1β* (*p* < 0.05), *IL-4Ra* (*p* < 0.05), and *IL-10* (*p* < 0.05) (Figure 1j–r). In addition, the microbial composition of FMT-treated mice tended to reprint MFGM-derived microbiota (Figure S1D-F) with an increased relative abundance of *Lactobacillus* (*p* < 0.001) and *unclassified_f__Muribaculaceae* (*p* < 0.01) and a decreased relative abundance of *Enterococcus* (*p* < 0.01) and *Escherichia-Shigella* (*p* < 0.01) at the genus level (Figure 1s,t). These results confirmed that MFGM-derived gut microbiota, especially *Lactobacillus* enrichment, are capable of improving maternal health and fetal development in IUGR pregnant mice.

### *Lactobacillus murinus* administration improved the litter weight and fetal development of IUGR pregnant mice

To identify the specific and dominant *Lactobacillus* specie responsible for the improvement of maternal–fetal outcomes, the representative OUTs of in the microbiota from MFGM-fed mice and FMT receipt mice were compared using BLAST. The results indicated that *L. murinus* was the only dominant and differential *Lactobacillus* specie with the highest abundance in IUGR pregnant mice, whether with or without MFGM supplementation, or following FMT with MFGM-derived microbiota (*p* < 0.01) (Figure 3a,b). Then, isolating a strain of *L. murinus* and administering it to IUGR pregnant mice (Figures 3c, S3) improved the litter characteristics by increasing litter weight (*p* < 0.05) and fetal weight (*p* < 0.05) without altering maternal body weight or litter size (*p* > 0.05) (Figure 3d–g). Meanwhile, IUGR-induced fetal intestinal barrier dysfunction and immune imbalance were greatly ameliorated by maternal *L. murinus* administration, evidenced by promoted intestinal morphology, and upregulated tight junctions of *Claudin-1* (*p* < 0.05), *Occludin* (*p* < 0.01), and *ZO-1* (*p* < 0.05) (Figure 3h–k). In addition, maternal *L. murinus* administration decreased CD11c^+^ pro-inflammatory macrophages (*p* < 0.05) without altering CD206^+^ anti-inflammatory macrophages (*p* > 0.05) in fetal intestine, along with immune cytokines expressions of *TNF-α* (*p* < 0.05), *IL-1β* (*p* < 0.05) and *IL-4Ra* (*p* < 0.01) (Figure 3l–t). These results confirmed that MFGM-enriched *L. murinus* improved maternal-fetal health outcomes.

### Intestinal and circulating SCFAs in IUGR pregnant mice were elevated by MFGM, MFGM-derived microbiota, or *L.* *murinus*

**Figure 4.**
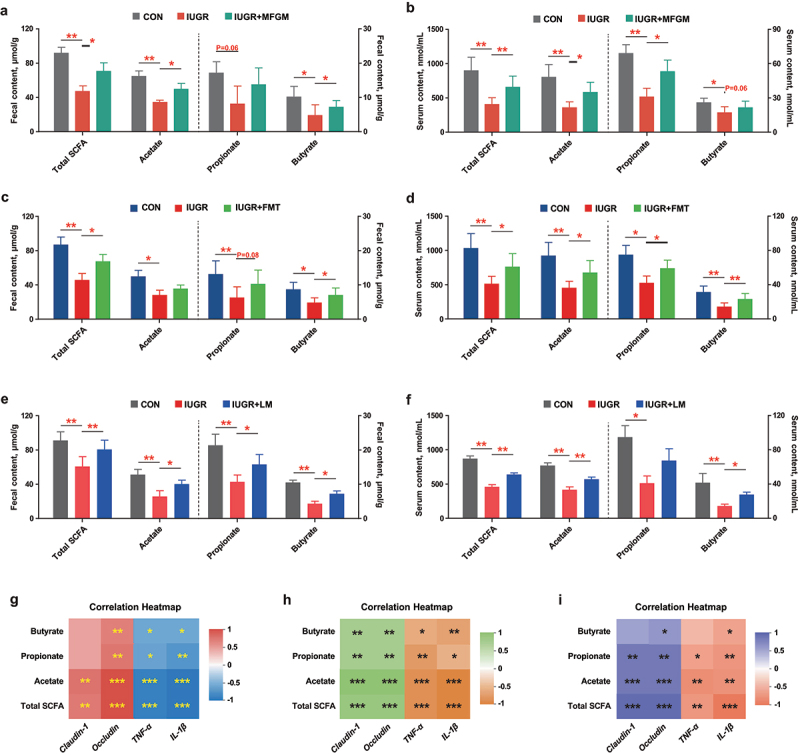
Intestinal and circulating SCFAs in IUGR pregnant mice were elevated by MFGM, MFGM-derived microbiota or *L.* *murinus*. Fecal and circulating SCFAs of pregnant mice with/without supplementation of MFGM (a, b), MFGM-derived fecal microbial suspension (c, d) or *L.* *murinus* (e, f); Spearman correlations of circulating SCFAs level and fetal intestinal parameters with/without supplementation of MFGM (g), MFGM-derived fecal microbial suspension (h) or *L.* *murinus* (i). *n* = 6; one-way ANOVA with Tukey’s test was used for statistical analysis. *p < 0.05, **p < 0.01, ***p < 0.001. CON, mice fed with normal protein diet from G0.5-G18.5; IUGR, mice fed with normal protein diet before G10.5 and low protein diet from G10.5-G18.5; IUGR + MFGM, mice fed with normal protein diet before G10.5 and low protein diet supplemented with MFGM from G10.5-G18.5; IUGR + FMT, mice fed with normal protein diet before G10.5 and low protein diet plus FMT with MFGM-derived fecal microbial suspension from G10.5-G18.5; IUGR + LM, mice fed with normal protein diet before G10.5 and low protein diet plus *L.* *murinus* administration from G10.5-G18.5; LM, *L.* *murinus*; FMT, fecal microbiota transplantation; SCFAs, short chain fatty acids. IRF4, interferon regulatory factor 4; IRF5, interferon regulatory factor 5; IL-10, interleukin-10; IL-4 Rα, interleukin-4 Rα; TNF-α, tumor necrosis factor-α; IL-1β, interleukin-1β; ZO-1, zonula occludens-1.

In this study, fecal and circulating SCFAs were reduced in IUGR pregnant mice, of which levels were mostly restored by supplementation with MFGM (Figure 4a, b), MFGM-derived microbiota (Figure 4c, d) or *L. murinus* (Figure 4e, f). The correlation analysis revealed that circulating SCFAs were positively related to fetal intestinal parameters (Figure 4g–i), suggesting that SCFAs might be the critical intermediate between gut microbiota and maternal–fetal development in IUGR pregnant mice.

### SCFAs cocktail supplementation improved litter weight and fetal development of IUGR pregnant mice

**Figure 5.**
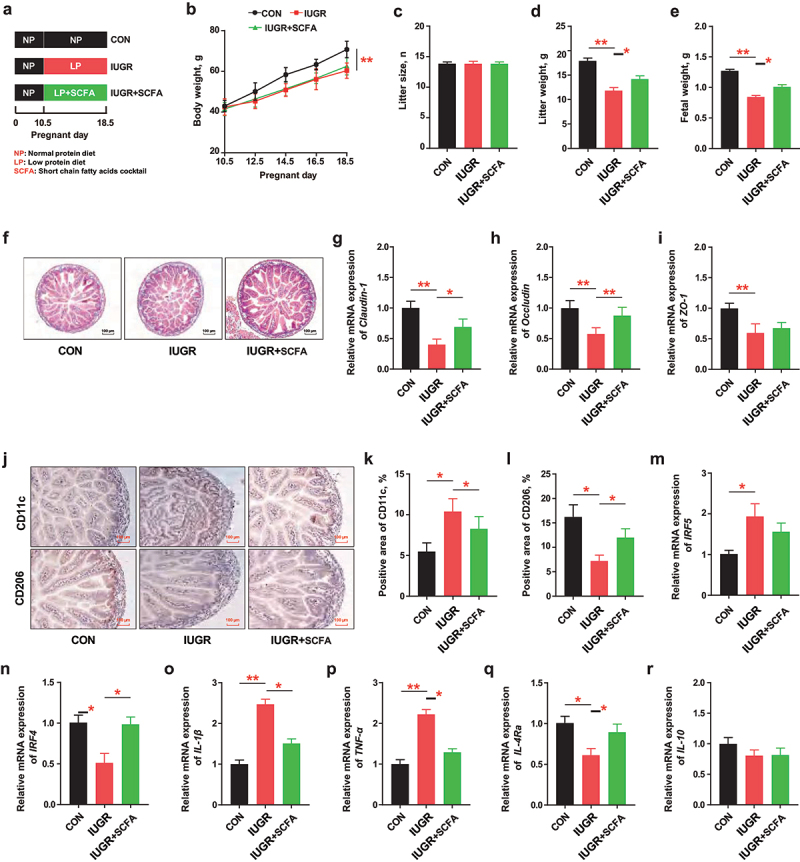
SCFAs cocktail supplementation improved litter weight and fetal development of IUGR pregnant mice. After mated, 8-week-old pregnant C57BL/6J mice (*n* = 6 per group) were fed with a normal protein diet (NP) before gestational day (GD) 10.5 and a low protein diet (LP) from GD10.5 to GD18.5 to induce intrauterine growth restriction (IUGR) and supplemented with/without SCFAs cocktail in the drinking water. (a) Study design; (b-e) body weight, litter size, litter weight and fetal weight of pregnant mice; (f-i) morphology and relative gene expressions of tight junctions Claudin-1, Occludin and ZO-1 of fetal jejunum; (j-n) immunohistochemical staining, percentage and relative gene expressions of biomarkers of M1/M2 macrophages in fetal jejunum; (o-r) relative gene expression of inflammatory cytokines of fetal jejunum; (s,t) fecal microbial analysis of community plots and differential microbiota of pregnant mice. *n* = 6; one-way ANOVA with Tukey’s test was used for statistical analysis of all other parameters. *p < 0.05, **p < 0.01, ***p < 0.001. CON, mice fed with normal protein diet from G0.5-G18.5; IUGR, mice fed with normal protein diet before G10.5 and low protein diet from G10.5-G18.5; IUGR + SCFA, mice fed with normal protein diet before G10.5 and low protein diet supplemented with SCFAs cocktail in the drinking water from G10.5-G18.5; IRF4, interferon regulatory factor 4; IRF5, interferon regulatory factor 5; IL-10, interleukin-10; IL-4 Rα, interleukin-4 Rα; tnf-α, tumor necrosis factor-α; IL-1β, interleukin-1β; ZO-1, zonula occludens-1; SCFAs, short chain fatty acids. IRF4, interferon regulatory factor 4; IRF5, interferon regulatory factor 5; IL-10, interleukin-10; IL-4 Rα, interleukin-4 Rα; TNF-α, tumor necrosis factor-α; IL-1β, interleukin-1β; ZO-1, zonula occludens-1.

To verify the role of SCFAs in promoting maternal outcomes and fetal development, exogenous SCFAs cocktail was directly supplemented to IUGR pregnant mice (Figure 5a). Results showed that SCFAs cocktail administration replicates the maternal-fetal phenotype observed in MFGM- or FMT-treated IUGR pregnant mice, by increasing the litter weight (*p* < 0.05) and fetal weight (*p* < 0.05) without altering the maternal body weight and litter size (*p* > 0.05) (Figure 5b–e). For IUGR fetuses, maternal SCFAs cocktail supplementation mitigated the intestinal injuries by enhancing barrier and immune functions with improved intestinal morphology, upregulated tight junctions of *Claudin-1* (*p* < 0.05) and *Occludin* (*p* < 0.01) (Figure 5f–i), decreased pro-inflammatory macrophages (*p* < 0.05), and increased anti-inflammatory macrophages (*p* < 0.05) with their biomarkers of *IRF4* (*p* < 0.05) and *IRF5* (*p* > 0.05), as well as cytokines expressions of *TNF-α* (*p* < 0.05), *IL-1β* (*p* < 0.05) and *IL-4Ra* (*p* < 0.05) (Figure 5j-r). These results confirmed that SCFAs, as the important MFGM-derived microbial metabolites, were greatly beneficial to improving maternal-fetal development in IUGR pregnant mice.

### MFGM, MFGM-derived microbiota, *L.* *murinus*, and SCFAs cocktail improved placental efficiency via activating PI3K-Akt pathway

**Figure 6.**
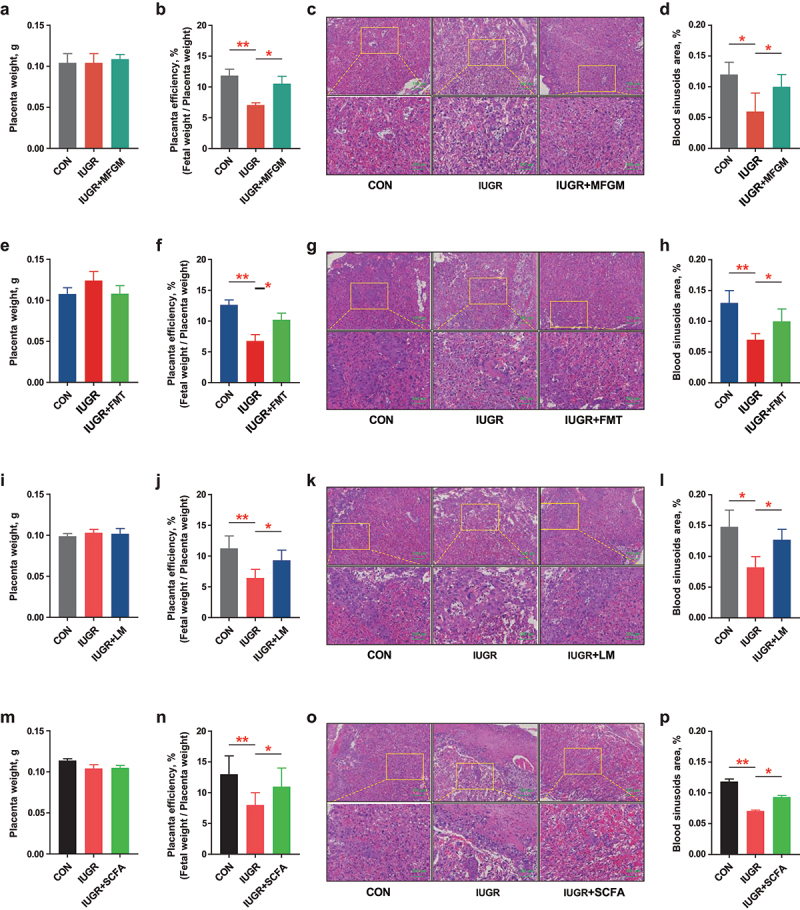
MFGM, MFGM-derived microbiota, *L. murinus* and SCFAs cocktails improved placenta efficiency of IUGR pregnant mice. Placenta weight, efficiency, H&E staining and blood sinusoids area of pregnant mice with/without supplementation of MFGM (a-d), MFGM-derived fecal microbial suspension (e-h), *L.* *murinus* (i-l), or SCFAs cocktail (m-p). *n* = 6; one-way ANOVA with Tukey’s test was used for statistical analysis. *p < 0.05, **p < 0.01, ***p < 0.001. CON, mice fed with normal protein diet from G0.5-G18.5; IUGR, mice fed with normal protein diet before G10.5 and low protein diet from G10.5-G18.5; IUGR + MFGM, mice fed with normal protein diet before G10.5 and low protein diet supplemented with MFGM from G10.5-G18.5; IUGR + FMT, mice fed with normal protein diet before G10.5 and low protein diet plus FMT with MFGM-derived fecal microbial suspension from G10.5-G18.5; IUGR + LM, mice fed with normal protein diet before G10.5 and low protein diet plus *L.* *murinus* administration from G10.5-G18.5; IUGR + SCFA, mice fed with normal protein diet before G10.5 and low protein diet supplemented with SCFAs cocktail in the drinking water from G10.5-G18.5; LM, *L.* *murinus*; FMT, fecal microbiota transplantation; SCFAs, short chain fatty acids.

In the present study, although the placental weight did not change (*p* > 0.05) (Figure 6a, e, i), the fetal/placental weight ratio was improved by MFGM (*p* < 0.05) (Figure 6b), MFGM-derived microbiota (*p* < 0.05) (Figure 6f), *L. murinus* (*p* < 0.05) (Figure 6j) or SCFAs cocktail (*p* < 0.05) (Figure 6n) in IUGR pregnant mice. The morphological observations showed that IUGR reduced placental sinusoid area, which was improved by administration of MFGM (*p* < 0.05) (Figure 6c, d), MFGM-derived microbiota (*p* < 0.05) (Figure 6g, h), *L. murinus* (*p* < 0.05) (Figure 6k, l) or SCFAs cocktail (*p* < 0.05) (Figure 6o, p). These results indicated that MFGM, MFGM-derived microbiota, dominant microbe *L. murinus*, and key metabolite SCFAs promoted maternal-fetal health by improving the placenta efficiency of IUGR pregnant mice.

**Figure 7.**
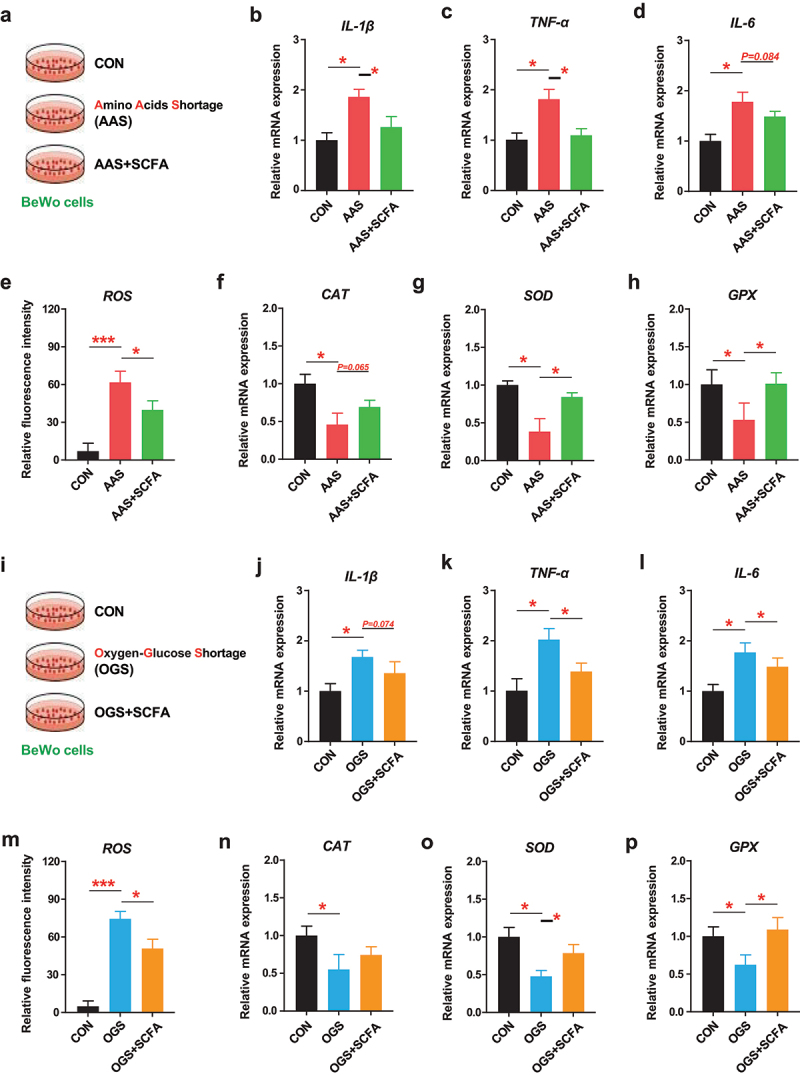
SCFAs cocktail promoted placental functions under amino acid shortage or oxygen-glucose shortage model. Amino acid shortage model (AAS) for placental BeWo cells was established by mixing basic and amino acid-free DMEM/F12 medium (7:1), and supplemented with/without SCFAs cocktail for 24 h. (a) Study design, (b-d) relative gene expressions of inflammatory cytokines, (e) relative fluorescence intensity of intracellular ROS, and (f-h) relative gene expressions of anti-oxidant parameters of SCFAs cocktail supplementation for placental cells under AAS model. Oxygen-glucose shortage model (OGS) for placental BeWo cells was established by mixing basic and glucose-free DMEM/F12 medium (7:1) in hypoxic conditions (94%N_2_/5%CO_2_/1%O_2_), and supplemented with/without SCFAs cocktail for 24 h. (i) Study design, (j-l) relative gene expressions of inflammatory cytokines, (m) relative fluorescence intensity of intracellular ROS, and (n-p) relative gene expressions of anti-oxidant parameters of SCFAs cocktail supplementation for placental cells under OGS model. *n* = 3; one-way ANOVA with Tukey’s test was used for statistical analysis. *p < 0.05, ***p < 0.001. CON, cells were treated with basic medium; AAS, cells were treated with amino acid shortage medium; OGS, cells were treated with oxygen-glucose shortage medium in hypoxic conditions; SCFA, short chain fatty acids cocktail. TNF-α, tumor necrosis factor-α; IL-1β, interleukin-1β; IL-6, interleukin-6; ROS, reactive oxygen species; CAT, catalase; SOD, superoxide dismutase; GPX, glutathione peroxidase.

To confirm the direct role of SCFAs cocktail in regulating placental functions, an amino acid shortage model (AAS) and an oxygen-glucose shortage model (OGS) for placental BeWo cells were established *in vitro* to mimic placental malnutrition in IUGR pregnant mice (Figure 7a, i). The results showed that SCFAs cocktail alleviated AAS- or OGS-induced placental inflammation and oxidative stress by downregulating *IL-1β* (*p* < 0.05; *p* = 0.074), *TNF-α* (*p* < 0.05; *p* < 0.05) and *IL-6* (*p* = 0.084; *p* < 0.05), reducing intracellular ROS (*p* < 0.05; *p* < 0.05), and upregulating *CAT* (*p* = 0.065; *p* > 0.05), *SOD* (*p* < 0.05; *p* < 0.05), and *GPX* (*p* < 0.05; *p* < 0.05) (Figure 7b-h, j-p). These results confirmed that MFGM-elevated circulating SCFAs directly improved the placental functions under the IUGR model.

**Figure 8.**
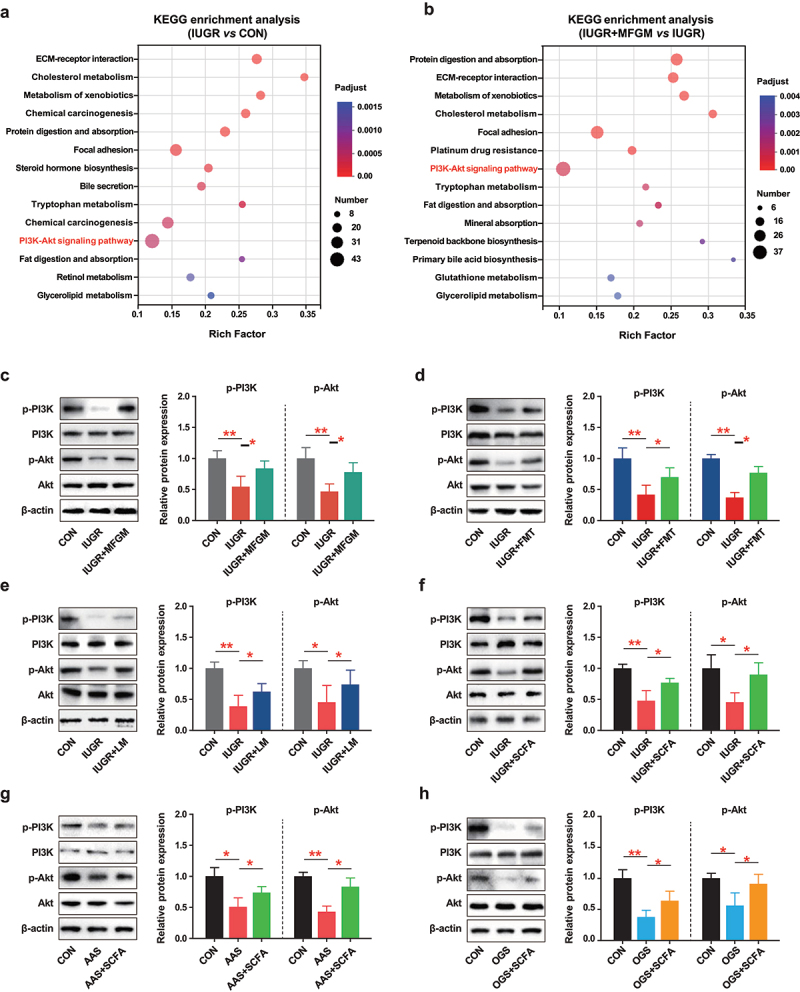
MFGM, MFGM-derived microbiota, *L. murinus* and SCFAs cocktails activated PI3K-Akt signaling in the placenta. KEGG enrichment analysis of placental differentially expressed genes (DEGs, , downregulation for IUGR *vs* CON; b, upregulation for IUGR + MFGM *vs* IUGR); relative phosphorylation of PI3K/Akt in IUGR placenta with/without supplementation of MFGM (c, *n* = 6), MFGM-derived fecal microbial suspension (d, *n* = 6), L. murinus (e, *n* = 6) or SCFAs cocktail (f, *n* = 6); relative phosphorylation of PI3K/Akt in placental cells under AAS (g, *n* = 3) or OGS (h, *n* = 3) model with/without SCFAs cocktail supplementation. One-way ANOVA with Tukey’s test was used for statistical analysis. *p < 0.05, **p < 0.01. CON, mice fed with normal protein diet from G0.5-G18.5 or cells were treated with basic medium; IUGR, mice fed with normal protein diet before G10.5 and low protein diet from G10.5-G18.5; IUGR + MFGM, mice fed with normal protein diet before G10.5 and low protein diet supplemented with MFGM from G10.5-G18.5; IUGR + FMT, mice fed with normal protein diet before G10.5 and low protein diet plus FMT with MFGM-derived fecal microbial suspension from G10.5-G18.5; IUGR + LM, mice fed with normal protein diet before G10.5 and low protein diet plus *L.* *murinus* administration from G10.5-G18.5; IUGR + SCFA, mice fed with normal protein diet before G10.5 and low protein diet supplemented with SCFAs cocktail in the drinking water from G10.5-G18.5; AAS, cells were treated with amino acid shortage medium; OGS, cells were treated with oxygen-glucose shortage medium in hypoxic conditions; LM, *L.* *murinus*; FMT, fecal microbiota transplantation; SCFAs, short chain fatty acids.

To study the underlying mechanisms of maternal MFGM intervention on the placenta functions, RNA-seq of placental tissues from IUGR pregnant mice with/without MFGM intervention was performed. The results indicated that most of the differentially expressed genes (DEGs) in the two groups were both annotated to PI3K/Akt signaling pathway by KEGG enrichment analysis (Figure 8a, b). In detail, the depressed phosphorylation of PI3K/Akt in IUGR placenta was restored by MFGM (*p* < 0.05) (Figure 8c), MFGM-derived microbiota (*p* < 0.05) (Figure 8d), *L. murinus* (*p* < 0.05) (Figure 8e) and SCFAs cocktail (*p* < 0.05) (Figure 8f). Furthermore, AAS- or OGS-induced depressed phosphorylation of PI3K/Akt was also activated by SCFAs cocktail supplementation in the culture medium (*p* < 0.05) (Figure 8g, h). These results indicated that PI3K/Akt signaling was activated by MFGM, MFGM-derived microbiota, *L. murinus*, and SCFAs cocktail in the process of improving placenta efficiency of IUGR pregnant mice.

To confirm the critical role of PI3K/Akt signaling in response to SCFAs cocktail supplementation, the dual inhibitor of PI3K/Akt, PI3K/AKT-IN-1, was applied to AAS and OGS models (Fig. S4 A, J). The results showed that when blocked by a specific inhibitor, the promoting effects and the activation of PI3K-Akt signaling by SCFAs cocktail were largely weakened with amino acid shortage (AAS)- or oxygen-glucose shortage (OGS)-treated placental cells, evidenced by slight changes of pro-inflammatory cytokines (*p* < 0.05) (Fig. S4 B-D, K-M) and anti-oxidant parameters (*p* < 0.05) (Fig. S4 E-H, N-Q). These results suggested that SCFAs cocktail promoted placental functions largely via activating PI3K/Akt signaling.

## Discussion

Intrauterine growth retardation (IUGR), or fetal growth restriction (FGR), is a serious reproductive issue that impairs fetal intestinal immune defense, villus morphology, and barrier function.^4,26^ Our previous findings suggested that maternal supplementation with milk fat globule membrane (MFGM) during gestation transitionally improved the intestinal development and growth performance of their offspring during the neonatal period.^18^ We hypothesized that the beneficial effects of gestational MFGM supplementation on neonates were attributable to the improved fetal growth and development. In this study, MFGM supplementation during gestation improved litter characteristics and enriched intestinal *L. murinus* to elevate intestinal and circulating SCFAs in IUGR pregnant mice. Besides, maternal MFGM-derived higher circulating SCFAs ameliorated maternal placental dysfunctions of oxidative stress and inflammation via activating PI3K/Akt signaling, thereby enhancing growth development of IUGR fetuses.

A reduced fetal weight for its gestational age is commonly defined as IUGR/FGR, leading to a birth weight less than the 10th percentile of predicted fetal weight.^27^ Consistent with previous study, reduced fetal weight and litter weight were observed in IUGR pregnant mice.^19^ These changes were largely reversed by supplementation with MFGM, MFGM-derived microbiota, *L. murinus*, or SCFAs cocktail for pregnant mice during gestation.^19^ For fetus, adaptive intestinal villi integrity and barrier functions are critical for nutrients absorption and defense against the invasion of pathogens after birth.^28,29^ Our previous studies illustrated that IUGR induced intestinal barrier dysfunctions, leading to a higher susceptibility to inflammation.^3,4^ Tight junctions are a major component of the intestinal barrier for maintaining the intestinal homeostasis and health.^30,31^ In the present study, the decreased gene expressions of intestinal tight junctions (*Claudin-1* and *Occludin*) in IUGR fetus were reversed by maternal supplementation with MFGM, MFGM-derived microbiota, *L. murinus*, or SCFAs cocktail, indicating the improved intestinal barrier functions in IUGR fetuses.^32^

Adequate placental efficiency and function are the chief regulator of nutrient supply and biological transition to the growing embryo during gestation.^33^ IUGR is frequently rooted in a dysfunctional placenta, characterized by reduced fetal/placental weight ratio and insufficient fetal growth.^34^ In the present study, the reduced fetal/placental weight ratio in IUGR mice was markedly reversed by supplementation with MFGM, MFGM-derived microbiota, *L. murinus*, or SCFAs cocktail, indicating improved placental efficiency and functions.^35^ In general, the loss in placental sinusoid area adversely affects placental nutrient transportation, which would be closely associated with the occurrence of IUGR.^36^ In this study, the reduced placental sinusoid area in IUGR mice was largely restored by maternal supplementation with MFGM, MFGM-derived microbiota, *L. murinus*, or SCFAs cocktail, suggesting a promoted maternal-placental-fetal nutrient transportation.^37^ Oxidative stress and inflammation in the placenta resulted in adverse fetal outcomes and higher neonatal susceptibility to illness and mortality after birth.^38^ In the present study, amino acids shortage model (AAS) and oxygen-glucose shortage model (OGS) were established to mimic malnutrition for placental cells, and oxidative stress and inflammation were found in AAS- or OGS-treated cells. Such adverse effects were reversed by SCFAs cocktail treatment, which confirmed a critical role of MFGM-elevated circulating SCFAs in the homeostasis of placental functions.^13^

SCFAs can activate G-coupled-receptors (GPR), inhibit histone deacetylases (HDAC), and serve as energy substrates, thus affecting various physiological processes and contributing to health and disease.^39^ It has been reported that SCFAs are typical ligands for GPR41, GPR43, and GPR109A, which can regulate energy metabolism and protect against inflammatory bowel disease.^40^ In addition, butyrate and propionate act as HDAC inhibitors and modulators of immune homeostasis, altering the expression of many genes with diverse functions, including cell proliferation, apoptosis, and differentiation.^41^ Previous studies showed that the PI3K/Akt signaling pathway was involved in the process of redox and maintaining inflammation homeostasis.^42,43^ In the present study, SCFAs cocktail improved IUGR-induced placental dysfunctions through PI3K/Akt pathway activation, which was in line with a previous study.^44^ Similarly, MFGM, MFGM-derived microbiota, or *L. murinus* also activated PI3K/Akt signaling and eventually promoted placental functions and fetal development, which provide insights into the comprehensive understanding of MFGM and SCFAs in physiological functions and maternal-placental-fetal health outcomes.

The gut-placenta-fetus axis connects the maternal gut, gut microbes, placenta, and fetus.^45,46^ Maternal microbial dysbiosis induces metabolic disturbance, inflammatory disorder, oxidative stress, and placental dysfunctions, consequently leading to IUGR or FGR.^47^ Depletion of the maternal gut microbiota restricts placental functions and impairs fetal growth.^48^ In the present study, *Lactobacillus*, especially *L. murinus*, which was dramatically decreased in the IUGR pregnant mice, was specifically enriched by MFGM supplementation and proved to modulate the intestinal microbial community and immune response, by which the maternal-fetal health outcomes were improved.^49^ Maternal microbiome-derived bioactive metabolites have been reported to promote maternal-placental-fetal circulation.^50^ In this study, microbial metabolites SCFAs decreased in the circulation of IUGR pregnant mice, while MFGM intervention or FMT with MFGM-derived microbiota could modulate gut microbial community by enriching *L. murinus* and other SCFAs-producing bacteria, such as *unclassified_f_Muribaclaceae*. These changes elevated the levels of luminal SCFAs which subsequently were penetrated into circulation, leading to an increased circulating SCFAs level. In agreement with a previous study which reported that gestational SCFAs supplementation prevented placental growth restriction and vascular insufficiency, our results indicated that SCFAs promoted the placental efficiency and functions in IUGR pregnant mice.^51^ Longitudinal fetal growth data indicated that fetal growth and weight gain mainly occur during late pregnancy. Poor second and third trimesters fetal growth has been associated with increased risks of preterm birth and low birthweight, and long-term adverse health outcomes.^52^ In this study, an IUGR pregnant mice model was established by feeding a low-protein diet during late gestation, as described previously.^29^ In consistency with this period, MFGM, as well as its-derived *L. murinus*, and SCFAs were supplemented during late gestation to explore their benefits for maternal-placental-fetal health outcomes.

Recent studies uncovered the direct bioactive effects of MFGM supplementation on neurodevelopment, cognitive function, gut microbiome, health, and metabolism in various animal models and infants.^53^ A randomized, control trial showed that adding bovine MFGM to the infant formula could modulate stool microbiota and elevate short chain fatty acids (SCFAs) in neonates.^54^ However, the effects of maternal MFGM supplementation on placental functions and fetal development, especially in IUGR condition, remained largely unexplored. This study revealed that gestational MFGM intervention modulated maternal gut microbiome by enriching *L. murinus*, which consequently elevated circulating SCFAs levels and promoted maternal-placental-fetal health outcomes. Although positive transitional effects of supplementation with MFGM, *L. murinus* and SCFAs have been found in IUGR pregnant mice model, further clinical trials are expected to examine the potential health-promoting effects on pregnant women, placental functions and fetal development with IUGR/FGR in the future.

## Conclusion

Taken together, MFGM supplementation during gestation improved the litter characteristics and enriched intestinal *L. murinus* to elevate luminal and circulating SCFAs in IUGR pregnant mice. Maternal elevated circulating SCFAs promoted IUGR-induced placental dysfunctions of oxidative stress and inflammation via activating PI3K/Akt signaling, eventually improving growth performance and intestinal development of IUGR fetuses. Our findings provide a new strategy for maternal–fetal nutrition in reducing IUGR-derived adverse outcomes.

## Supplementary Material

supplementary_materials clean.docx

## Data Availability

The 16S rRNA amplicon sequencing data, genome sequencing data of *L. murinus* and RNA sequencing data have also been deposited in the NCBI Sequence Read Archive (SRA) repository under BioProject PRJNA1142212.
