# Supplementary material for: Maternal milk fat globule membrane enriched gut L. murinus and circulating SCFAs to improve placental efficiency and fetal development in intrauterine growth restricted mice model
Source: Gut Microbes. 2025 Jan 6;17(1):2449095. doi: 10.1080/19490976.2024.2449095 (PMC12931695; doi:10.1080/19490976.2024.2449095)
Supplement: supplementary_materials clean.docx [file KGMI_A_2449095_SM3563.docx]

**Supplementary Table 1. Nutritional compositions of normal protein diet and low protein diet**

| **Ingredients** | **Normal protein** | **Low protein** |
| --- | --- | --- |
| Corn starch, % | 41.30 | 54.30 |
| Casein, % | 19.00 | 8.00 |
| Maltodextrin, % | 13.20 | 13.20 |
| Sucrose, % | 10.00 | 10.00 |
| Soybean oil, % | 7.00 | 5.00 |
| Cellulose, % | 5.00 | 5.00 |
| Mineral premix^1^, % | 3.50 | 3.50 |
| Vitamin premix^2^, % | 1.00 | 1.00 |
| Total | 100.00 | 100.00 |
| Nutritional level^3^ |  |  |
| Protein, % | 19.00 | 8.00 |
| Carbohydrate, % | 61.00 | 71.00 |
| Lipids, % | 7.50 | 5.60 |
| Fiber, % | 3.00 | 3.60 |
| Ash, % | 5.50 | 4.10 |
| Calcium, % | 1.00 | 0.90 |
| Phosphorus, % | 0.78 | 0.76 |
| Water, % | 4.72 | 6.04 |
| Total energy, kcal/kg | 3616 | 3664 |

^1^ Mineral premix contains (per kg): Ca 142.94 g, K 102.81 g, Na 29.11 g, P 44.61 g, Cl, 44.89 g, Mg 14.48 g, S 8.57 g, Fe 1.00 g, Zn 860.31 g, Si 143.26 mg, Mn 301.08 mg, Cu 172.41 mg, Cr 28.66 mg, B 14.26 mg, F 28.73 mg, Ni 14.31 mg, Li 2.85 mg, Se 4.28 mg, I 5.93 mg, Mo 4.32 mg, V 2.87 mg.

^2^ Vitamin premix contains (per kg): Nicotinic acid 3.00 g, Ca pantothenate 1.60 g, Pyridoxine-HCl 0.70 g, Thlamin-HCl 0.60 g, Riboflavin 0.60 g, Folic acid 0.20 g, Biotin 20 mg, Vitamin B_12_ 2.5 g, Vitamin E 7,500 IU, Vitamin A 400,000 IU, Vitamin D_3_ 100,000 IU, Vitamin K_1_ 75 mg.

^3^ Calculated level. The standard specification of nutritional level: Protein ≥18% (normal protein), Carbohydrate ≥55%, Lipids ≥4.0%, Fiber ≤5.0%, Ash ≤8.0%, Calcium 0.8%-1.2%, Phosphorus 0.6%-1.2%, Water ≤10.0%, Total energy ≥3600 kcal/kg.

**Supplementary Table 2. Nutritional compositions of MFGM**

| **Compositions** | **Content** | **Specification** | **Method** |
| --- | --- | --- | --- |
| Protein (%) | 70.0 | >66.0 | AOAC |
| Phospholipid (%) | 6.0 | >4.5 | AOAC |
| Fat (%) | 15.0 | <20.0 | AOAC |
| Lactose (%) | 4.0 | <8.0 | AOAC |
| Moisture (%) | 4.5 | <6.0 | AOAC |
| Ash (%) | 3.0 | <4.0 | AOAC |
| Scorched Particles (mg/25g) | 7 | <7.5 | SMEDP |

AOAC, Association of Official Analytical Chemists; SMEDP, Standard Methods for the Examination of Dairy Products.

**Supplementary Table 3. The primer sequences for qRT-PCR**

| Genes | Primer | Sequence (5' to 3') | Size, bp | Accession No. |
| --- | --- | --- | --- | --- |
| *β-actin* | Forward | CGTTGACATCCGTAAAGACC | 281 | NM_007393 |
|  | Reverse | AACAGTCCGCCTAGAAGCAC |  |  |
| *IRF4* | Forward | GAACGAGGAGAAGAGCGTCTTC | 184 | NM_013674 |
|  | Reverse | GTAGGAGGATCTGGCTTGTCGA |  |  |
| *IRF5* | Forward | GGTCAACGGGGAAAAGAAACT | 119 | NM_001252382.1 |
|  | Reverse | CATCCACCCCTTCAGTGTACT |  |  |
| *IL-10* | Forward | GCTCTTACTGACTGGCATGAG | 105 | NM_007393 |
|  | Reverse | CGCAGCTCTAGGAGCATGTG |  |  |
| *IL-4Ra* | Forward | TCTGCATCCCGTTGTTTTGC | 245 | NM_001008700.4 |
|  | Reverse | GCACCTGTGCATCCTGAATG |  |  |
| *TNF-α* | Forward | CCCTCACACTCAGATCATCTTCT | 61 | NM_013693 |
|  | Reverse | GCTACGACGTGGGCTACAG |  |  |
| *IL-1β* | Forward | GCAACTGTTCCTGAACTCAACT | 89 | NM_008361.3 |
|  | Reverse | ATCTTTTGGGGTCCGTCAACT |  |  |
| *Claudin-1* | Forward | AGCTGTGCATGGCCTCTTGT | 162 | NM_001163574.2 |
|  | Reverse | CCAATGTCAATGGCAACACCCT |  |  |
| *Occludin* | Forward | CAGCCTCGGTACAGCAGCAAT | 78 | NM_008756.2 |
|  | Reverse | ATAGTGGTCAGGGTCCGTCCTC |  |  |
| *ZO-1* | Forward | CGGAACTATGACCATCGCCTAC | 186 | NM_001163574.1 |
|  | Reverse | CTTCGGGATGTTGTCTGGAGTC |  |  |

IRF4, interferon regulatory factor 4; IRF5, interferon regulatory factor 5; IL-10, interleukin-10; IL-4Rα, interleukin-4Rα; TNF-α, tumor necrosis factor-α; IL-1β, interleukin-1β; ZO-1, zonula occludens-1


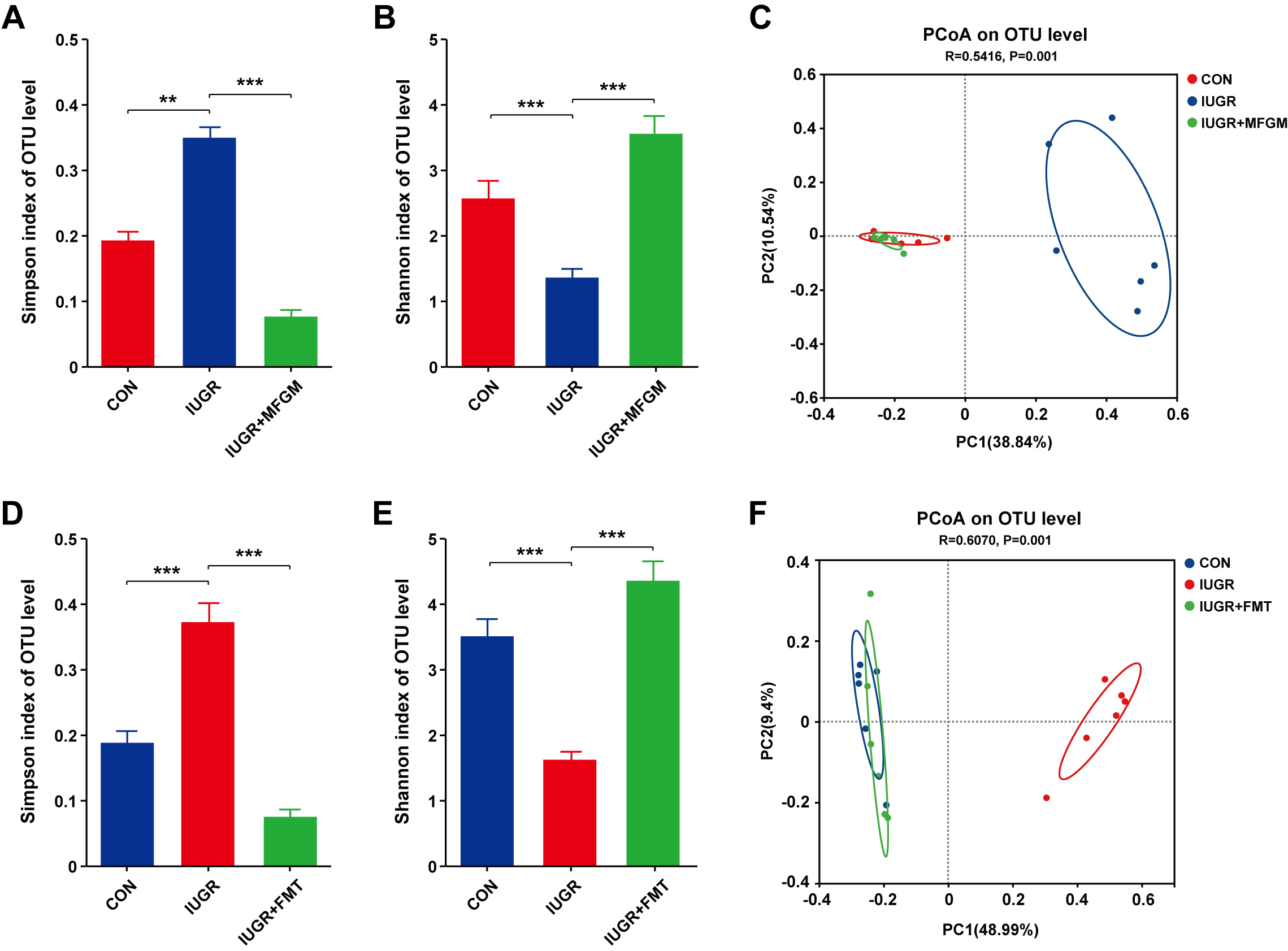


**Fig S1. Microbial α-diversity and PCoA analysis of pregnant mice supplemented with/without MFGM or FMT with MFGM-derived fecal suspension**

Microbial α-diversity (Simpson index and Shannon index) and principal coordinate analysis (PCoA) analysis of IUGR mice supplemented with/without MFGM (**A-C**) or FMT with MFGM-derived fecal suspension (**D-F**). PCoA analysis was based on unweighted unifrac distance. The Kruskal-Wallis test and post-hoc Tukey-Kramer test was used for microbial analysis. n = 6, ** *P* < 0.01, *** *P* < 0.001. OUT, operational taxonomic units; CON, Mice fed with normal protein diet from G 0.5-G 18.5; IUGR, Mice fed with normal protein diet before G10.5 and low protein diet from G 10.5-G 18.5; IUGR + MFGM, Mice fed with normal protein diet before G10.5 and low protein diet supplemented with MFGM from G 10.5-G 18.5; IUGR + FMT, Mice fed with normal protein diet before G10.5 and low protein diet plus FMT with MFGM-derived fecal microbial suspension from G 10.5-G 18.5.

**
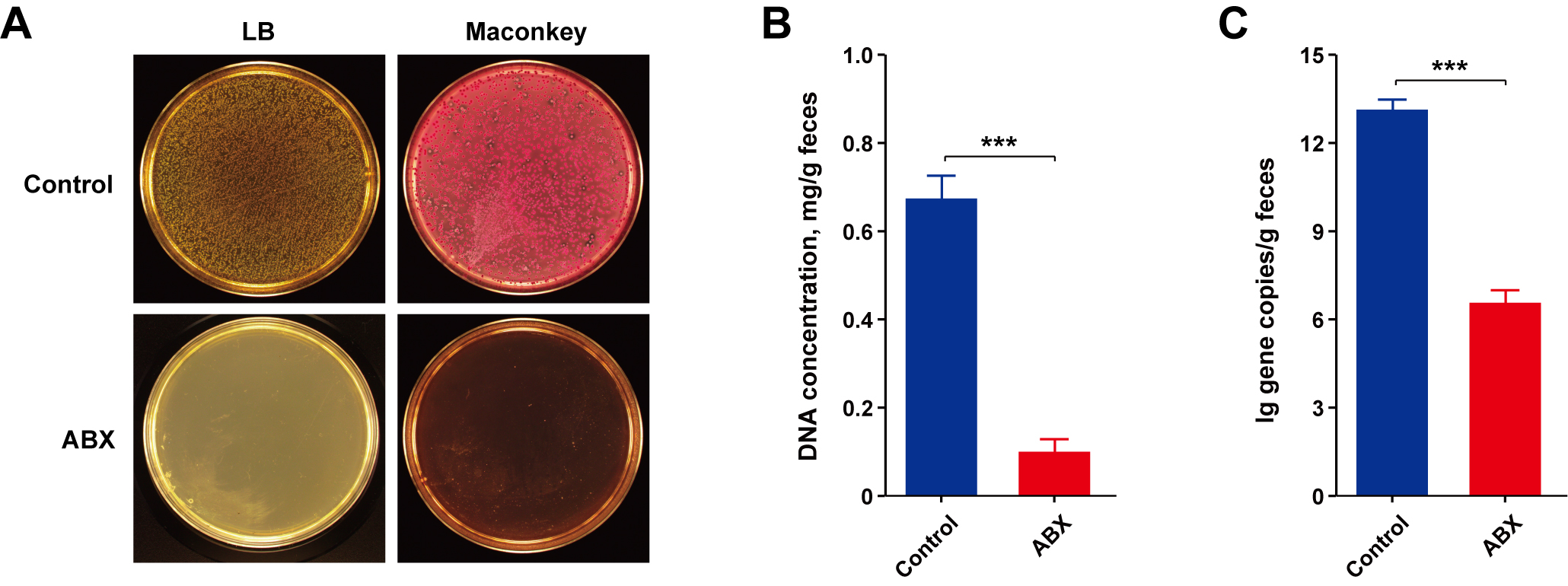
**

**Fig S2. Verification of intesitnal microbiota depletion**

Fecal microbiota culture on LB and Maconkey agar (**A**), fecal DNA concentration (**B**), and genome copies of total bacteria (**C**) in female mice before mating or treated with antibiotic cocktail after mating for 10.5 days. The Student’s t test was used for analysis. n = 6, *** *P* < 0.001. LB, Luria-Bertani agar; Maconkey, Maconkey agar; Control, female mice were fed with normal water before mating; ABX, pregnant mice were fed with antibiotic cocktail water for 10.5 days after mating.


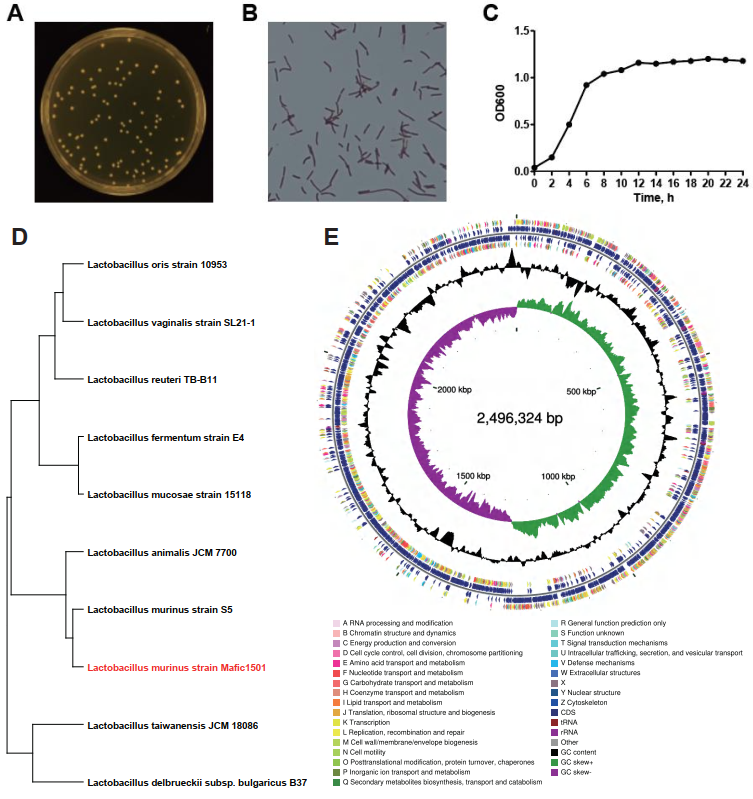


**Fig S3. Background of *L. murinus***

The colony mrophplogy (A), gram staining (B), growth curve (C), phylogenetic tree (D) and genome sequence (E) of *L. murinus*.


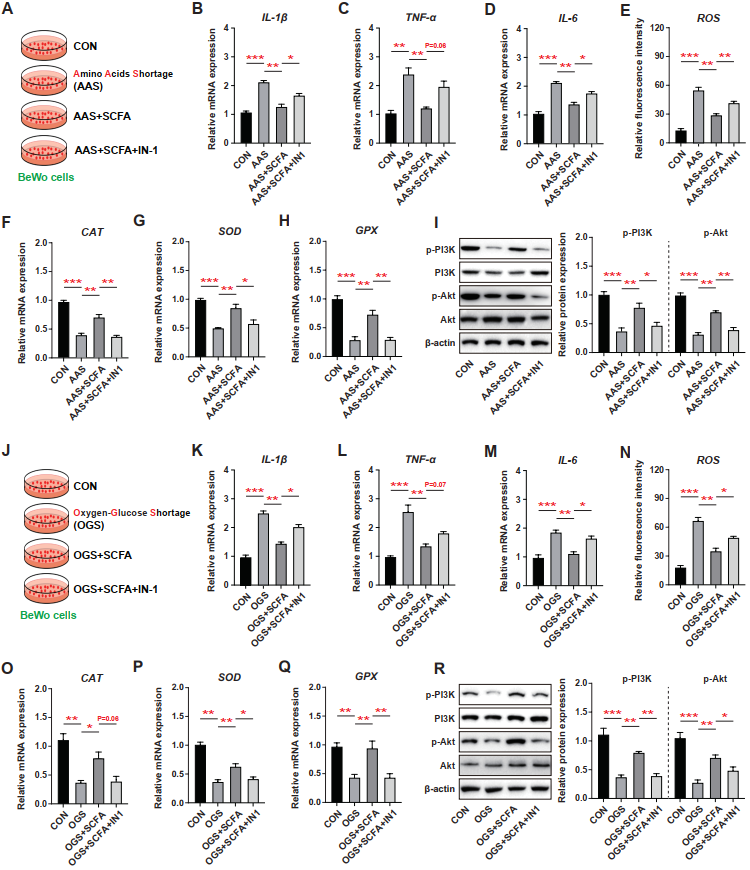


**Fig S4. SCFAs cocktail promoted placental functions via PI3K/Akt signaling under amino acid shortage or oxygen-glucose shortage model**

Amino acid shortage model (AAS) for placental BeWo cells was established by mixing basic and amino acid-free DMEM/F12 medium (7:1), and supplemented with/without SCFAs cocktail and/or PI3K/Akt inhibitor (IN1) for 24 h. (**A**) Study design, (**B-D**) relative gene expressions of inflammatory cytokines, (**E**) relative fluorescence intensity of intracellular ROS, and (**F-H**) relative gene expressions of anti-oxidant parameters of SCFAs cocktail and/or IN1 supplementation for placental cells under AAS model. Oxygen-glucose shortage model (OGS) for placental BeWo cells were established by mixing basic and glucose-free DMEM/F12 medium (7:1) in hypoxic conditions (94%N_2_ / 5%CO_2_ / 1%O_2_), and supplemented with/without SCFAs cocktail and/or PI3K/Akt inhibitor (IN1) for 24 h. (**I**) Study design, (**J-L**) relative gene expressions of inflammatory cytokines, (**M**) relative fluorescence intensity of intracellular ROS, and (**N-P**) relative gene expressions of anti-oxidant parameters of SCFAs cocktail and/or PI3K/Akt inhibitor (IN1) supplementation for placental cells under OGS model. n = 3; one-way ANOVA with Tukey’s test was used for statistical analysis. * *P* < 0.05; ** *P* < 0.01; *** *P* < 0.001. CON, cells were treated with basic medium; AAS, cells were treated with amino acid shortage medium; OGS, cells were treated with oxygen-glucose shortage medium in hypoxic conditions; SCFA, short chain fatty acids cocktail. TNF-α, tumor necrosis factor-α; IL-1β, interleukin-1β; IL-6, interleukin-6; ROS, reactive oxygen species; CAT, catalase; SOD, superoxide dismutase; GPX, glutathion peroxidase.
